# 4-Chloro-*N*-[(*E*)-(3,4-dimeth­oxy­phen­yl)methyl­idene]aniline

**DOI:** 10.1107/S1600536810032782

**Published:** 2010-08-18

**Authors:** M. Nawaz Tahir, Muhammad Ilyas Tariq, Muhammad Sarfraz, Shahbaz Ahmad, Riaz H. Tariq

**Affiliations:** a Department of Physics, University of Sargodha, Sargodha, Pakistan; b Department of Chemistry, University of Sargodha, Sargodha, Pakistan; c Institute of Chemical and Pharmaceutical Sciences, The University of Faisalabad, Faisalabad, Pakistan

## Abstract

The asymmetric unit of the title compound, C_15_H_14_ClNO_2_, contains two mol­ecules with significantly different conformations: the dihedral angles between the 4-chloro­aniline and 3,4-dimeth­oxy­phenyl (excluding C atoms) moieties are 19.68 (7) and 45.54 (4)°. In the crystal, the mol­ecules are linked by C—H⋯O hydrogen bonds and weak C—H⋯π inter­actions.

## Related literature

For related structures, see: Dehno Khalaji *et al.* (2009 ▶); Shang & Tan (2007 ▶).
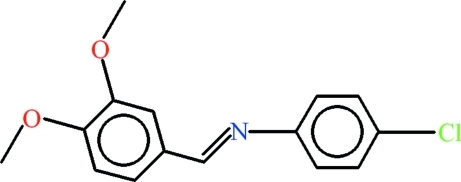


## Experimental

### 

#### Crystal data

C_15_H_14_ClNO_2_
*M* *_r_* = 275.72
Monoclinic, 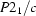

*a* = 12.4227 (4) Å
*b* = 7.3638 (2) Å
*c* = 30.4583 (13) Å
β = 96.080 (2)°
*V* = 2770.60 (17) Å^3^
*Z* = 8
Mo *K*α radiation
μ = 0.27 mm^−1^
*T* = 296 K
0.35 × 0.22 × 0.20 mm

#### Data collection

Bruker Kappa APEXII CCD diffractometer
Absorption correction: multi-scan (*SADABS*; Bruker, 2005 ▶) *T*_min_ = 0.932, *T*_max_ = 0.950
20975 measured reflections
5007 independent reflections
3456 reflections with *I* > 2σ(*I*)
*R*_int_ = 0.026

#### Refinement

*R*[*F* ^2^ > 2σ(*F* ^2^)] = 0.040
*wR*(*F* ^2^) = 0.120
*S* = 1.05
5007 reflections
347 parameters
H-atom parameters constrained
Δρ_max_ = 0.19 e Å^−3^
Δρ_min_ = −0.30 e Å^−3^

### 

Data collection: *APEX2* (Bruker, 2009 ▶); cell refinement: *SAINT* (Bruker, 2009 ▶); data reduction: *SAINT*; program(s) used to solve structure: *SHELXS97* (Sheldrick, 2008 ▶); program(s) used to refine structure: *SHELXL97* (Sheldrick, 2008 ▶); molecular graphics: *ORTEP-3 for Windows* (Farrugia, 1997 ▶) and *PLATON* (Spek, 2009 ▶); software used to prepare material for publication: *WinGX* (Farrugia, 1999 ▶) and *PLATON*.

## Supplementary Material

Crystal structure: contains datablocks global, I. DOI: 10.1107/S1600536810032782/hb5610sup1.cif

Structure factors: contains datablocks I. DOI: 10.1107/S1600536810032782/hb5610Isup2.hkl

Additional supplementary materials:  crystallographic information; 3D view; checkCIF report

## Figures and Tables

**Table 1:** Hydrogen-bond geometry (Å, °) *Cg*1, *Cg*2 and *Cg*3 are the centroids of the C1–C6, C8–C13 and C16–C21 rings, respectively.

*D*—H⋯*A*	*D*—H	H⋯*A*	*D*⋯*A*	*D*—H⋯*A*
C14—H14*B*⋯O3^i^	0.96	2.52	3.468 (3)	170
C6—H6⋯*Cg*3^ii^	0.93	2.85	3.602 (2)	139
C18—H18⋯*Cg*1	0.93	2.89	3.588 (2)	133
C21—H21⋯*Cg*3^iii^	0.93	2.88	3.549 (2)	130
C29—H29*C*⋯*Cg*2	0.96	2.88	3.782 (2)	157
C30—H30*C*⋯*Cg*1^iv^	0.96	2.76	3.613 (2)	148

Symmetry codes: (i) 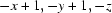; (ii) 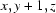; (iii) 
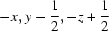
; (iv) 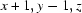.

**Fig. 1.**
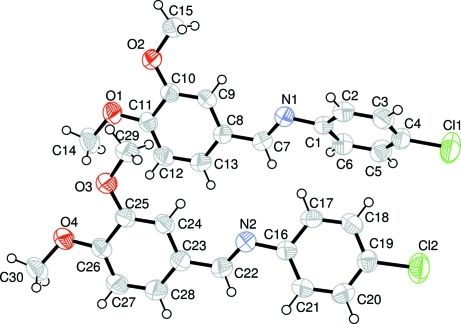
View of (I) with displacement ellipsoids drawn at the 50% probability level. H-atoms are shown by small circles of arbitrary radii.

## supplementary crystallographic information

### Comment

The crystal structures of (II) i.e, 4-chloro-*N*-(3,4,5-trimethoxybenzylidene)aniline (Dehno Khalaji *et al.*, 2009) and (III) i.e, 4-[(4-chlorophenyl)iminomethyl]-2-methoxyphenol (Shang & Tan, 2007) have been published which are related to the title compound (I, Fig. 1)

The title compound consists of two molecules in the crystallographic asymmetric unit which differ from each other geometrically. In one molecule, the 4-chloroanilinic group A (C1—C6/N1/CL1) and group B (C7—C13/O1/O2) of 3,4-dimethoxyphenyl are planar with r.m.s. deviation of 0.0081 and 0.0146 Å, respectively. The dihedral angle between A/B is 45.54 (4)°. The C-atoms C14 and C15 of 3,4-dimethoxyphenyl are at a distance of -0.0058 (35) and 0.1132 (34) Å, respectively from the parent group B. In the second molecule, the 4-chloroanilinic group C (C16—C21/N2/CL2) and group D (C22—C28/O3/O4) of 3,4-dimethoxyphenyl are planar with r.m.s. deviation of 0.0094 and 0.0063 Å, respectively. The dihedral angle between C/D is 19.68 (7)°. The C-atoms C29 and C30 of 3,4-dimethoxyphenyl are at a distance of -0.2256 (33) and -0.2205 (31) Å, respectively from the parent group D. This shows that both molecules differ at large from each other. The molecules are stabilized through C—H···O type of H-bonding and π···π interactions (Table 1).

### Experimental

Equimolar quantities of 4-chloroaniline and 3,4-dimethoxybenzaldehyde were refluxed in methanol for 30 min. The solution was kept at room temperature which afforded orange light yellow prisms of (I) after 48 h.

### Refinement

The H-atoms were positioned geometrically (C–H = 0.93–0.96 Å) and refined as riding with *U*_iso_(H) = *xU*_eq_(C), where *x* = 1.5 for methyl and *x* = 1.2 for other H-atoms.

### Crystal data

**Table:**

C_15_H_14_ClNO_2_	*F*(000) = 1152
*M**_r_* = 275.72	*D*_x_ = 1.322 Mg m^−^^3^
Monoclinic, *P*2_1_/*c*	Mo *K*α radiation, λ = 0.71073 Å
Hall symbol: -P 2ybc	Cell parameters from 3456 reflections
*a* = 12.4227 (4) Å	θ = 1.7–25.3°
*b* = 7.3638 (2) Å	µ = 0.27 mm^−^^1^
*c* = 30.4583 (13) Å	*T* = 296 K
β = 96.080 (2)°	Prism, light yellow
*V* = 2770.60 (17) Å^3^	0.35 × 0.22 × 0.20 mm
*Z* = 8	

### Data collection

**Table:**

Bruker Kappa APEXII CCD diffractometer	5007 independent reflections
Radiation source: fine-focus sealed tube	3456 reflections with *I* > 2σ(*I*)
graphite	*R*_int_ = 0.026
Detector resolution: 8.10 pixels mm^-1^	θ_max_ = 25.3°, θ_min_ = 1.7°
ω scans	*h* = −14→14
Absorption correction: multi-scan (*SADABS*; Bruker, 2005)	*k* = −8→8
*T*_min_ = 0.932, *T*_max_ = 0.950	*l* = −36→29
20975 measured reflections	

### Refinement

**Table:**

Refinement on *F*^2^	Primary atom site location: structure-invariant direct methods
Least-squares matrix: full	Secondary atom site location: difference Fourier map
*R*[*F*^2^ > 2σ(*F*^2^)] = 0.040	Hydrogen site location: inferred from neighbouring sites
*wR*(*F*^2^) = 0.120	H-atom parameters constrained
*S* = 1.05	*w* = 1/[σ^2^(*F*_o_^2^) + (0.0465*P*)^2^ + 1.0036*P*] where *P* = (*F*_o_^2^ + 2*F*_c_^2^)/3
5007 reflections	(Δ/σ)_max_ < 0.001
347 parameters	Δρ_max_ = 0.19 e Å^−^^3^
0 restraints	Δρ_min_ = −0.30 e Å^−^^3^

### Special details

**Table:**

Geometry. Bond distances, angles *etc*. have been calculated using the rounded fractional coordinates. All su's are estimated from the variances of the (full) variance-covariance matrix. The cell e.s.d.'s are taken into account in the estimation of distances, angles and torsion angles
Refinement. Refinement of *F*^2^ against ALL reflections. The weighted *R*-factor *wR* and goodness of fit *S* are based on *F*^2^, conventional *R*-factors *R* are based on *F*, with *F* set to zero for negative *F*^2^. The threshold expression of *F*^2^ > σ(*F*^2^) is used only for calculating *R*-factors(gt) *etc*. and is not relevant to the choice of reflections for refinement. *R*-factors based on *F*^2^ are statistically about twice as large as those based on *F*, and *R*- factors based on ALL data will be even larger.

### Fractional atomic coordinates and isotropic or equivalent isotropic displacement parameters (Å^2^)

**Table:**

	*x*	*y*	*z*	*U*_iso_*/*U*_eq_	
Cl1	−0.47381 (6)	0.59742 (12)	0.19551 (3)	0.1029 (3)	
O1	0.34541 (11)	0.7300 (2)	0.00839 (5)	0.0666 (6)	
O2	0.15540 (11)	0.7756 (2)	−0.03006 (4)	0.0622 (5)	
N1	−0.10335 (14)	0.6680 (2)	0.09001 (5)	0.0531 (6)	
C1	−0.18906 (15)	0.6522 (3)	0.11683 (6)	0.0480 (6)	
C2	−0.27949 (16)	0.5526 (3)	0.10049 (7)	0.0534 (7)	
C3	−0.36655 (17)	0.5352 (3)	0.12478 (7)	0.0582 (8)	
C4	−0.36430 (18)	0.6199 (3)	0.16498 (8)	0.0597 (8)	
C5	−0.27691 (19)	0.7213 (3)	0.18141 (7)	0.0641 (8)	
C6	−0.18948 (17)	0.7378 (3)	0.15726 (7)	0.0572 (8)	
C7	−0.00599 (17)	0.6629 (3)	0.10774 (7)	0.0547 (7)	
C8	0.08663 (16)	0.6815 (3)	0.08263 (7)	0.0505 (7)	
C9	0.07247 (15)	0.7174 (3)	0.03730 (6)	0.0481 (7)	
C10	0.15987 (16)	0.7355 (3)	0.01380 (6)	0.0477 (6)	
C11	0.26563 (16)	0.7137 (3)	0.03515 (7)	0.0505 (7)	
C12	0.27930 (17)	0.6779 (3)	0.07967 (7)	0.0595 (8)	
C13	0.19067 (17)	0.6630 (3)	0.10334 (7)	0.0606 (8)	
C14	0.45431 (17)	0.7121 (4)	0.02793 (9)	0.0797 (10)	
C15	0.05181 (18)	0.8106 (4)	−0.05280 (7)	0.0704 (9)	
Cl2	−0.28854 (6)	0.13333 (12)	0.26687 (2)	0.0916 (3)	
O3	0.34354 (11)	0.2151 (2)	0.04035 (5)	0.0685 (6)	
O4	0.53216 (11)	0.1383 (2)	0.07674 (5)	0.0614 (5)	
N2	0.08863 (14)	0.1523 (2)	0.16357 (6)	0.0538 (6)	
C16	0.00252 (16)	0.1438 (3)	0.19048 (6)	0.0474 (7)	
C17	−0.09141 (16)	0.2363 (3)	0.17535 (7)	0.0526 (7)	
C18	−0.18002 (17)	0.2347 (3)	0.19879 (7)	0.0575 (7)	
C19	−0.17623 (17)	0.1391 (3)	0.23760 (7)	0.0535 (7)	
C20	−0.08492 (18)	0.0463 (3)	0.25324 (7)	0.0614 (8)	
C21	0.00371 (17)	0.0481 (3)	0.22977 (7)	0.0572 (7)	
C22	0.18473 (17)	0.1189 (3)	0.17922 (7)	0.0597 (8)	
C23	0.27594 (16)	0.1226 (3)	0.15321 (7)	0.0547 (7)	
C24	0.26207 (16)	0.1687 (3)	0.10854 (7)	0.0511 (7)	
C25	0.34811 (16)	0.1725 (3)	0.08394 (7)	0.0502 (7)	
C26	0.45269 (15)	0.1286 (3)	0.10403 (7)	0.0515 (7)	
C27	0.46640 (17)	0.0814 (3)	0.14774 (8)	0.0654 (8)	
C28	0.37878 (18)	0.0795 (4)	0.17212 (8)	0.0700 (9)	
C29	0.23950 (17)	0.2317 (3)	0.01634 (7)	0.0672 (8)	
C30	0.63635 (16)	0.0663 (3)	0.09192 (8)	0.0675 (9)	
H2	−0.28136	0.49722	0.07297	0.0640*	
H3	−0.42638	0.46651	0.11397	0.0699*	
H5	−0.27641	0.77864	0.20863	0.0769*	
H6	−0.13016	0.80729	0.16828	0.0686*	
H7	0.00653	0.64637	0.13813	0.0656*	
H9	0.00291	0.72905	0.02296	0.0577*	
H12	0.34870	0.66362	0.09400	0.0714*	
H13	0.20112	0.64022	0.13353	0.0727*	
H14A	0.46754	0.79754	0.05169	0.1193*	
H14B	0.50294	0.73565	0.00611	0.1193*	
H14C	0.46581	0.59102	0.03914	0.1193*	
H15A	0.00931	0.70152	−0.05403	0.1056*	
H15B	0.05986	0.85091	−0.08225	0.1056*	
H15C	0.01616	0.90321	−0.03750	0.1056*	
H17	−0.09447	0.30033	0.14893	0.0631*	
H18	−0.24229	0.29817	0.18840	0.0690*	
H20	−0.08271	−0.01779	0.27963	0.0737*	
H21	0.06554	−0.01593	0.24041	0.0687*	
H22	0.19776	0.09042	0.20906	0.0716*	
H24	0.19328	0.19723	0.09526	0.0614*	
H27	0.53481	0.05060	0.16104	0.0785*	
H28	0.38922	0.04869	0.20187	0.0840*	
H29A	0.19869	0.12289	0.01982	0.1007*	
H29B	0.24729	0.24997	−0.01437	0.1007*	
H29C	0.20224	0.33350	0.02736	0.1007*	
H30A	0.66641	0.13300	0.11741	0.1012*	
H30B	0.68330	0.07689	0.06896	0.1012*	
H30C	0.62931	−0.05923	0.09960	0.1012*	

### Atomic displacement parameters (Å^2^)

**Table:**

	*U*^11^	*U*^22^	*U*^33^	*U*^12^	*U*^13^	*U*^23^
Cl1	0.0819 (5)	0.1266 (7)	0.1086 (6)	0.0040 (4)	0.0499 (4)	0.0131 (5)
O1	0.0419 (8)	0.0932 (12)	0.0652 (10)	−0.0014 (8)	0.0074 (7)	0.0011 (9)
O2	0.0502 (8)	0.0884 (11)	0.0482 (8)	0.0024 (8)	0.0058 (7)	0.0031 (8)
N1	0.0510 (10)	0.0576 (10)	0.0509 (10)	−0.0004 (8)	0.0067 (8)	0.0022 (8)
C1	0.0490 (11)	0.0469 (11)	0.0480 (11)	0.0033 (9)	0.0048 (9)	0.0062 (9)
C2	0.0576 (13)	0.0507 (12)	0.0512 (12)	0.0006 (10)	0.0028 (10)	0.0015 (10)
C3	0.0496 (12)	0.0575 (13)	0.0664 (14)	−0.0020 (10)	0.0005 (10)	0.0109 (11)
C4	0.0568 (13)	0.0607 (14)	0.0640 (14)	0.0086 (11)	0.0179 (11)	0.0117 (11)
C5	0.0749 (16)	0.0635 (14)	0.0554 (13)	0.0048 (12)	0.0139 (12)	−0.0054 (11)
C6	0.0574 (13)	0.0566 (13)	0.0572 (13)	−0.0035 (10)	0.0045 (10)	−0.0052 (10)
C7	0.0574 (13)	0.0604 (13)	0.0464 (11)	0.0015 (10)	0.0059 (10)	0.0051 (10)
C8	0.0492 (12)	0.0535 (12)	0.0487 (11)	0.0007 (9)	0.0043 (9)	0.0026 (9)
C9	0.0419 (11)	0.0496 (12)	0.0518 (12)	0.0011 (9)	0.0001 (9)	−0.0002 (9)
C10	0.0482 (11)	0.0469 (11)	0.0472 (11)	0.0005 (9)	0.0015 (9)	−0.0022 (9)
C11	0.0450 (11)	0.0514 (12)	0.0548 (12)	−0.0009 (9)	0.0043 (9)	−0.0033 (10)
C12	0.0438 (11)	0.0724 (15)	0.0601 (14)	0.0015 (10)	−0.0049 (10)	−0.0011 (11)
C13	0.0588 (13)	0.0740 (15)	0.0478 (12)	0.0017 (11)	0.0000 (10)	0.0064 (11)
C14	0.0445 (13)	0.113 (2)	0.0814 (17)	−0.0016 (13)	0.0054 (12)	−0.0137 (16)
C15	0.0656 (15)	0.0966 (18)	0.0477 (12)	0.0126 (13)	0.0000 (11)	−0.0030 (12)
Cl2	0.0734 (4)	0.1239 (6)	0.0828 (5)	0.0051 (4)	0.0329 (3)	−0.0075 (4)
O3	0.0441 (8)	0.1016 (12)	0.0596 (9)	0.0077 (8)	0.0045 (7)	0.0180 (9)
O4	0.0406 (8)	0.0748 (10)	0.0686 (10)	0.0043 (7)	0.0045 (7)	0.0094 (8)
N2	0.0529 (10)	0.0587 (11)	0.0503 (10)	0.0024 (8)	0.0072 (8)	0.0034 (8)
C16	0.0516 (12)	0.0447 (11)	0.0452 (11)	0.0001 (9)	0.0023 (9)	−0.0007 (9)
C17	0.0593 (13)	0.0538 (12)	0.0432 (11)	0.0071 (10)	−0.0019 (9)	0.0047 (9)
C18	0.0528 (12)	0.0617 (13)	0.0562 (13)	0.0120 (10)	−0.0030 (10)	−0.0051 (11)
C19	0.0538 (12)	0.0565 (12)	0.0509 (12)	0.0000 (10)	0.0094 (10)	−0.0085 (10)
C20	0.0689 (15)	0.0616 (14)	0.0543 (13)	0.0024 (11)	0.0092 (11)	0.0119 (11)
C21	0.0549 (12)	0.0573 (13)	0.0586 (13)	0.0091 (10)	0.0021 (10)	0.0126 (11)
C22	0.0588 (14)	0.0723 (15)	0.0474 (12)	0.0005 (11)	0.0035 (10)	0.0030 (11)
C23	0.0500 (12)	0.0606 (13)	0.0531 (12)	0.0010 (10)	0.0031 (10)	0.0013 (10)
C24	0.0425 (11)	0.0537 (12)	0.0564 (12)	0.0045 (9)	0.0016 (9)	0.0031 (10)
C25	0.0471 (11)	0.0496 (12)	0.0531 (12)	0.0015 (9)	0.0011 (9)	0.0050 (10)
C26	0.0423 (11)	0.0512 (12)	0.0600 (13)	0.0006 (9)	0.0005 (9)	0.0018 (10)
C27	0.0440 (12)	0.0853 (17)	0.0642 (14)	0.0044 (11)	−0.0073 (10)	0.0105 (12)
C28	0.0580 (14)	0.0963 (18)	0.0538 (13)	0.0041 (12)	−0.0028 (11)	0.0113 (13)
C29	0.0576 (13)	0.0810 (16)	0.0606 (14)	0.0047 (12)	−0.0046 (11)	0.0085 (12)
C30	0.0416 (12)	0.0733 (15)	0.0867 (17)	0.0067 (10)	0.0029 (11)	0.0070 (13)

### Geometric parameters (Å, °)

**Table:**

Cl1—C4	1.735 (2)	C13—H13	0.9300
Cl2—C19	1.734 (2)	C14—H14A	0.9600
O1—C11	1.354 (2)	C14—H14B	0.9600
O1—C14	1.425 (3)	C14—H14C	0.9600
O2—C15	1.419 (3)	C15—H15A	0.9600
O2—C10	1.364 (2)	C15—H15B	0.9600
O3—C25	1.360 (3)	C15—H15C	0.9600
O3—C29	1.421 (3)	C16—C17	1.387 (3)
O4—C30	1.430 (3)	C16—C21	1.388 (3)
O4—C26	1.358 (2)	C17—C18	1.374 (3)
N1—C7	1.272 (3)	C18—C19	1.372 (3)
N1—C1	1.414 (2)	C19—C20	1.366 (3)
N2—C22	1.262 (3)	C20—C21	1.375 (3)
N2—C16	1.416 (3)	C22—C23	1.450 (3)
C1—C6	1.384 (3)	C23—C24	1.395 (3)
C1—C2	1.389 (3)	C23—C28	1.382 (3)
C2—C3	1.380 (3)	C24—C25	1.369 (3)
C3—C4	1.372 (3)	C25—C26	1.414 (3)
C4—C5	1.368 (3)	C26—C27	1.369 (3)
C5—C6	1.380 (3)	C27—C28	1.381 (3)
C7—C8	1.454 (3)	C17—H17	0.9300
C8—C9	1.398 (3)	C18—H18	0.9300
C8—C13	1.384 (3)	C20—H20	0.9300
C9—C10	1.369 (3)	C21—H21	0.9300
C10—C11	1.412 (3)	C22—H22	0.9300
C11—C12	1.374 (3)	C24—H24	0.9300
C12—C13	1.383 (3)	C27—H27	0.9300
C2—H2	0.9300	C28—H28	0.9300
C3—H3	0.9300	C29—H29A	0.9600
C5—H5	0.9300	C29—H29B	0.9600
C6—H6	0.9300	C29—H29C	0.9600
C7—H7	0.9300	C30—H30A	0.9600
C9—H9	0.9300	C30—H30B	0.9600
C12—H12	0.9300	C30—H30C	0.9600
			
C11—O1—C14	117.62 (17)	H15A—C15—H15B	109.00
C10—O2—C15	117.28 (15)	O2—C15—H15A	109.00
C25—O3—C29	117.64 (16)	O2—C15—H15B	109.00
C26—O4—C30	118.33 (17)	N2—C16—C17	116.51 (17)
C1—N1—C7	119.52 (17)	N2—C16—C21	125.58 (19)
C16—N2—C22	121.01 (18)	C17—C16—C21	117.89 (19)
N1—C1—C6	123.42 (18)	C16—C17—C18	121.0 (2)
N1—C1—C2	117.81 (17)	C17—C18—C19	119.7 (2)
C2—C1—C6	118.70 (18)	Cl2—C19—C18	119.96 (17)
C1—C2—C3	120.48 (19)	Cl2—C19—C20	119.48 (17)
C2—C3—C4	119.6 (2)	C18—C19—C20	120.6 (2)
C3—C4—C5	121.0 (2)	C19—C20—C21	119.7 (2)
Cl1—C4—C3	119.47 (17)	C16—C21—C20	121.2 (2)
Cl1—C4—C5	119.53 (18)	N2—C22—C23	123.6 (2)
C4—C5—C6	119.5 (2)	C22—C23—C24	120.96 (18)
C1—C6—C5	120.8 (2)	C22—C23—C28	120.6 (2)
N1—C7—C8	122.94 (19)	C24—C23—C28	118.41 (19)
C7—C8—C9	120.89 (18)	C23—C24—C25	121.05 (19)
C7—C8—C13	120.26 (19)	O3—C25—C24	125.80 (19)
C9—C8—C13	118.85 (19)	O3—C25—C26	114.64 (17)
C8—C9—C10	120.72 (18)	C24—C25—C26	119.57 (19)
O2—C10—C11	114.53 (17)	O4—C26—C25	114.67 (18)
O2—C10—C9	125.53 (18)	O4—C26—C27	125.80 (18)
C9—C10—C11	119.93 (17)	C25—C26—C27	119.54 (19)
O1—C11—C10	114.68 (18)	C26—C27—C28	120.1 (2)
O1—C11—C12	126.11 (19)	C23—C28—C27	121.3 (2)
C10—C11—C12	119.21 (19)	C16—C17—H17	119.00
C11—C12—C13	120.5 (2)	C18—C17—H17	119.00
C8—C13—C12	120.8 (2)	C17—C18—H18	120.00
C1—C2—H2	120.00	C19—C18—H18	120.00
C3—C2—H2	120.00	C19—C20—H20	120.00
C2—C3—H3	120.00	C21—C20—H20	120.00
C4—C3—H3	120.00	C16—C21—H21	119.00
C4—C5—H5	120.00	C20—C21—H21	119.00
C6—C5—H5	120.00	N2—C22—H22	118.00
C1—C6—H6	120.00	C23—C22—H22	118.00
C5—C6—H6	120.00	C23—C24—H24	119.00
C8—C7—H7	119.00	C25—C24—H24	119.00
N1—C7—H7	119.00	C26—C27—H27	120.00
C8—C9—H9	120.00	C28—C27—H27	120.00
C10—C9—H9	120.00	C23—C28—H28	119.00
C11—C12—H12	120.00	C27—C28—H28	119.00
C13—C12—H12	120.00	O3—C29—H29A	109.00
C12—C13—H13	120.00	O3—C29—H29B	109.00
C8—C13—H13	120.00	O3—C29—H29C	109.00
O1—C14—H14B	109.00	H29A—C29—H29B	109.00
H14A—C14—H14C	109.00	H29A—C29—H29C	109.00
O1—C14—H14C	109.00	H29B—C29—H29C	109.00
H14A—C14—H14B	109.00	O4—C30—H30A	109.00
O1—C14—H14A	109.00	O4—C30—H30B	109.00
H14B—C14—H14C	109.00	O4—C30—H30C	109.00
O2—C15—H15C	109.00	H30A—C30—H30B	109.00
H15A—C15—H15C	109.00	H30A—C30—H30C	109.00
H15B—C15—H15C	109.00	H30B—C30—H30C	110.00
			
C14—O1—C11—C10	179.1 (2)	O2—C10—C11—O1	−2.1 (3)
C14—O1—C11—C12	−1.3 (3)	O2—C10—C11—C12	178.25 (19)
C15—O2—C10—C9	3.4 (3)	C9—C10—C11—O1	178.4 (2)
C15—O2—C10—C11	−176.1 (2)	C9—C10—C11—C12	−1.3 (3)
C29—O3—C25—C26	169.99 (18)	O1—C11—C12—C13	−179.5 (2)
C29—O3—C25—C24	−9.7 (3)	C10—C11—C12—C13	0.1 (3)
C30—O4—C26—C25	−169.27 (18)	C11—C12—C13—C8	0.8 (3)
C30—O4—C26—C27	10.8 (3)	N2—C16—C17—C18	179.24 (19)
C1—N1—C7—C8	−178.84 (19)	C21—C16—C17—C18	0.7 (3)
C7—N1—C1—C6	41.7 (3)	N2—C16—C21—C20	−179.1 (2)
C7—N1—C1—C2	−141.6 (2)	C17—C16—C21—C20	−0.6 (3)
C22—N2—C16—C21	−21.7 (3)	C16—C17—C18—C19	−0.5 (3)
C16—N2—C22—C23	179.0 (2)	C17—C18—C19—Cl2	−178.91 (17)
C22—N2—C16—C17	159.9 (2)	C17—C18—C19—C20	0.3 (3)
N1—C1—C6—C5	178.2 (2)	Cl2—C19—C20—C21	178.95 (17)
C6—C1—C2—C3	−1.9 (3)	C18—C19—C20—C21	−0.3 (3)
N1—C1—C2—C3	−178.78 (19)	C19—C20—C21—C16	0.5 (3)
C2—C1—C6—C5	1.5 (3)	N2—C22—C23—C24	1.6 (3)
C1—C2—C3—C4	1.2 (3)	N2—C22—C23—C28	−178.1 (2)
C2—C3—C4—Cl1	−179.88 (17)	C22—C23—C24—C25	179.9 (2)
C2—C3—C4—C5	−0.1 (3)	C28—C23—C24—C25	−0.4 (3)
Cl1—C4—C5—C6	179.46 (17)	C22—C23—C28—C27	179.6 (2)
C3—C4—C5—C6	−0.3 (3)	C24—C23—C28—C27	0.0 (4)
C4—C5—C6—C1	−0.4 (3)	C23—C24—C25—O3	179.9 (2)
N1—C7—C8—C9	3.9 (3)	C23—C24—C25—C26	0.2 (3)
N1—C7—C8—C13	−175.8 (2)	O3—C25—C26—O4	0.8 (3)
C7—C8—C9—C10	179.8 (2)	O3—C25—C26—C27	−179.21 (19)
C9—C8—C13—C12	−0.6 (3)	C24—C25—C26—O4	−179.42 (19)
C13—C8—C9—C10	−0.5 (3)	C24—C25—C26—C27	0.5 (3)
C7—C8—C13—C12	179.1 (2)	O4—C26—C27—C28	179.0 (2)
C8—C9—C10—C11	1.5 (3)	C25—C26—C27—C28	−1.0 (3)
C8—C9—C10—O2	−178.0 (2)	C26—C27—C28—C23	0.7 (4)

### Hydrogen-bond geometry (Å, °)

**Table:**

Cg1, Cg2 and Cg3 are the centroids of the C1–C6, C8–C13 and C16–C21 rings, respectively.

**Table:**

*D*—H···*A*	*D*—H	H···*A*	*D*···*A*	*D*—H···*A*
C14—H14B···O3^i^	0.96	2.52	3.468 (3)	170
C6—H6···Cg3^ii^	0.93	2.85	3.602 (2)	139
C18—H18···Cg1	0.93	2.89	3.588 (2)	133
C21—H21···Cg3^iii^	0.93	2.88	3.549 (2)	130
C29—H29C···Cg2	0.96	2.88	3.782 (2)	157
C30—H30C···Cg1^iv^	0.96	2.76	3.613 (2)	148

Symmetry codes: (i) −*x*+1, −*y*+1, −*z*; (ii) *x*, *y*+1, *z*;  (iii) −*x*, *y*−1/2, −*z*+1/2; (iv) *x*+1, *y*−1, *z*.
